# Fabrication of a Solution-Processed White Light Emitting Diode Containing a Single Dimeric Copper(I) Emitter Featuring Combined TADF and Phosphorescence

**DOI:** 10.3390/mi12121500

**Published:** 2021-11-30

**Authors:** Gang Cheng, Dongling Zhou, Uwe Monkowius, Hartmut Yersin

**Affiliations:** 1 State Key Laboratory of Synthetic Chemistry, Department of Chemistry, The University of Hong Kong, Pokfulam Road, Hong Kong, China; dlzhou@connect.hku.hk; 2 Hong Kong Quantum AI Lab Limited, 17 Science Park West Avenue, Pak Shek Kok, Hong Kong, China; 3 HKU Shenzhen Institute of Research and Innovation, Shenzhen 518053, China; 4 School of Education, Chemistry, Johannes Kepler University Linz, Altenbergerstr. 69, A-4040 Linz, Austria; 5 Institut für Physikalische Chemie, Universität Regensburg, D-93053 Regensburg, Germany

**Keywords:** dimeric copper(I) complexes, P∩N phosphine ligands, combined thermally activated delayed fluorescence and phosphorescence, thermally activated delayed fluorescence (TADF), combined singlet and triplet harvesting, organic light emitting diodes (OLEDs), white emission, white light emitting OLED (WOLED)

## Abstract

Luminescent copper(I) complexes showing thermally activated delayed fluorescence (TADF) have developed to attractive emitter materials for organic light emitting diodes (OLEDs). Here, we study the brightly luminescent dimer Cu_2_Cl_2_(P∩N)_2_ (P∩N = diphenylphosphanyl-6-methyl-pyridine), which shows both TADF and phosphorescence at ambient temperature. A solution-processed OLED with a device structure ITO/PEDOT:PSS/PYD2: Cu_2_Cl_2_(P∩N)_2_/DPEPO (10 nm)/TPBi (40 nm)/LiF (1.2 nm)/Al (100 nm) shows warm white emission with moderate external quantum efficiency (EQE). Methods for EQE increase strategies are discussed.

## 1. Introduction

Emerging new display and lighting technologies have considerably stimulated research efforts in the development of new luminescent materials. Organic light emitting diodes (OLEDs) use tailored emitter molecules, which efficiently harvest both singlet and triplet excitons formed in the emission layer at a ratio of 1:3 due to simple spin statistic considerations [1]. It was recognized early that phosphorescent heavy/noble metal complexes can fulfill this requirement because of efficient intersystem crossing (ISC) processes facilitated by the high spin-orbit coupling (SOC) induced by the metal center. This well-established strategy is known as triplet harvesting, because the emission stems from the lowest triplet state T_1_. It has been shown that OLEDs using such complexes can exploit up to 100% of all formed excitons leading to 100% internal quantum efficiency (IQE) [2,3,4,5,6]. Nevertheless, this concept comes with the prize of using expensive and rare noble metals like iridium or platinum. As only a very small amount of these noble metals per m^2^ display area are needed for modern OLEDs, the price per one display unit is unimportant and therefore, use of these metals seems to be acceptable. However, just because of the low noble metal content, recycling of the metal is either not possible or economically not viable. Therefore, one must assume that these metals are irretrievably lost and will be evenly distributed among the Earth’s ecosystem as with many other valuable (or persistent) materials of widely used consumer products. As soon as the OLED technology will dominate the display and lighting area, even such small amounts will sum up to a considerable consumption of these precious metals, which thus would be lost for other important applications, such as catalytic processes in the chemical industry [7].

Therefore, similar to the trend in catalysis, increasing research activities are focused on the replacement of noble metals by non-precious and abundant metals or even by purely organic compounds while still maintaining a high efficiency of the OLEDs [8]. The related exciton issue is addressed by designing emitter molecules with small singlet-triplet splittings ∆E(S_1_ − T_1_), being one essential condition for fast and efficient reverse intersystem crossing (rISC) T_1_ → S_1_ from the triplet state T_1_ to the excited singlet state S_1_ which ideally shows efficient S_1_ → S_0_ fluorescence. Although long known as thermally activated delayed fluorescence (TADF) or *E*-type fluorescence [9], use of this process for OLEDs was proposed only in 2006/2008 by Yersin et al. [10] and was designated as *singlet harvesting*, because the luminescence of the TADF emitters stems from the lowest excited singlet state. For the first time, this mechanism was exploited for OLED application in 2010 [11]. Besides other metals, particularly copper(I) complexes have proven to feature efficient TADF due to the distinct metal-to-ligand charge transfer (MLCT) character of their emissive states [12,13,14,15,16,17,18,19,20,21,22,23,24,25,26,27,28,29,30,31,32]. Additionally, a large number of purely organic compounds have been investigated, especially, by the Adachi group [33,34,35,36,37,38]. These molecules feature required photophysical properties, such as high emission quantum yields and relatively fast emission decay times lying in the range of a few µs and can lead to high external quantum efficiencies (EQE) in OLEDs [39,40,41,42,43,44]. Very recently, almost “zero-gap compounds” have been presented that also allow for 100% exciton use, based on a mechanism that is designated as *direct singlet harvesting* [45,46]. Using this mechanism, that is, applying the specifically designed molecules as OLED emitters, sub-micro second decay times can easily be achieved [45].

It has been shown that emissive copper(I) complexes sometimes do not only emit via the TADF process, but additionally feature phosphorescence [25,47,48,49,50,51,52,53,54,55,56,57,58,59,60,61,62]. The Cu(I) materials applied for many of these investigations are based on a patent filed almost one decade ago [63]. This combination of singlet and triplet emission shortens the emission decay time. These complexes can be regarded as both singlet and triplet harvesting materials, and thus are interesting for application in OLEDs. Indeed, corresponding device studies have already been reported [64,65].

**Figure 1:**
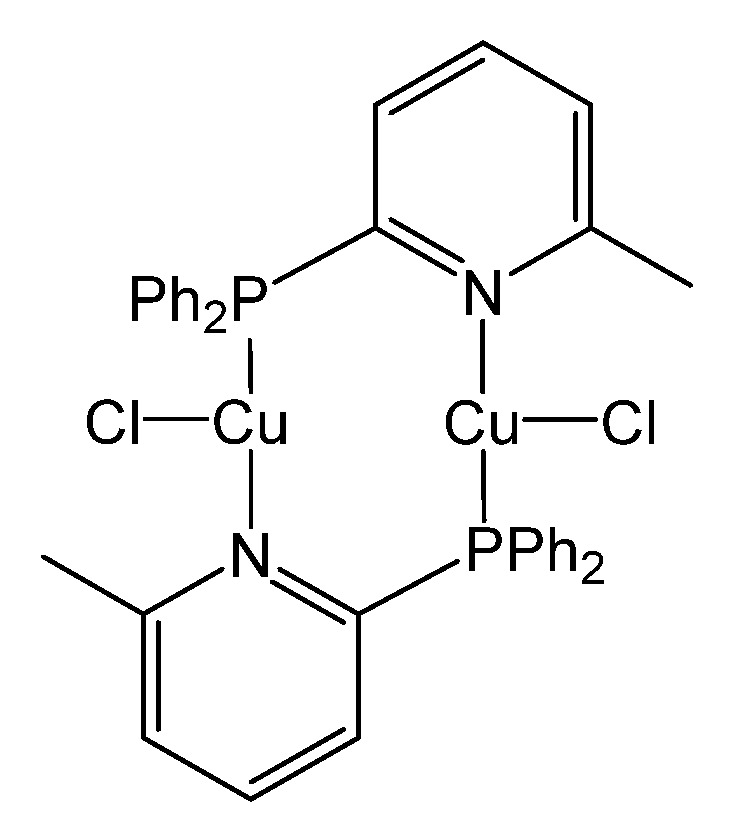
Di-nuclear Cu(I) complex, Cu_2_Cl_2_(P∩N)_2_ featuring both TADF and phosphorescence [48].

In recent studies, we have presented a series of di-nuclear copper(I) complexes with bridging P∩N phosphane ligands (whereas the nitrogen atom is part of a pyridine-type moiety) and discussed their photophysical properties in detail [47,48,49,50]. One of these materials, Cu_2_Cl_2_(P∩N)_2_ (Figure 1), shows remarkably high emission quantum yield at a moderate emission decay time (φ_PL_ = 92%, τ = 8.3 µs) even at ambient temperature as powder material. This compound was not yet investigated in opto-electronic devices. Accordingly, it is the subject of this investigation to study the compound’s properties in solution-processed OLEDs, and also to contribute to a better understanding of such Cu(I) based devices. Interestingly, its broad emission spectrum enables us to fabricate white light emitting OLEDs (WOLEDs) by using Cu_2_Cl_2_(P∩N)_2_ as a single emitter. Compared to those manufactured with multiple emitters, WOLEDs with a single emitter have the merits of easy fabrication, low cost, and, more importantly, of avoiding the issue of color aging [66].

## 2. Photophysical Background of Cu_2_Cl_2_(P∩N)_2_

Cu_2_Cl_2_(P∩N)_2_ was synthesized as described in ref. [48]. It represents one of the first compounds for which an ambient temperature emission has been reported that consists of combined phosphorescence and TADF [48]. In particular, the material shows ≈20% direct T_1_ → S_0_ phosphorescence and ≈80% delayed S_1_ → S_0_ fluorescence. This is a consequence of the relatively high spin-orbit coupling (SOC) experienced by the T_1_ state. The efficiency of SOC is also displayed in a large zero-field splitting of this triplet state [48, and compare [67]. Both states, T_1_ and S_1_, stem from the HOMO → LUMO transition of metal-to-ligand charge transfer (MLCT) character. Hence, the states represent ^1^MLCT(S_1_) and ^3^MLCT(T_1_) states. Both are in fast thermal equilibrium at ambient temperature. Due to the occurrence of two separate decay paths with decay rates of k(T_1_ → S_0_, phosphorescence) = 2.4 × 10^4^ s^−1^ (formally 42 µs) and k(S_1_ → S_0_, TADF) = 9.1 × 10^4^ s^−1^ (formally 11 µs), the overall decay time is reduced from the TADF-only decay time of 11 to 8.3 µs [48].

As neat powder, the material shows an emission quantum yield of Φ_PL_ = 92% with an emission peak maximum at λ_max_ = 485 nm. However, if doped with 8 wt % in di(9*H*-carbazol-9-yl)pyridine (PYD2) (see next section), Φ_PL_ decreases to 27% (in PMMA (poly(methyl methacrylate)) to ≈8%) and the peak maximum red-shifts to 544 nm (Table 1). Such a behavior is frequently found for Cu(I) complexes and is ascribed to a flattening distortion in the excited MLCT state [68,69,70]. This process is connected with an energy stabilization of the excited states. Accordingly, the emission is red shifted. Besides, a polarity change of the emitter environment might also play a role [45,71]. It has been shown that the flattening processes are less distinct in rigid crystalline environments than in softer matrices, such as many polymers or PYD2 [14,51]. Moreover, the geometry distortion in the excited states usually results in larger Franck-Condon (FC) factors between the lower energy vibrational wavefunctions of the involved excited state with higher energy vibrational wavefunctions of the ground state. These FC factors strongly govern the non-radiative decay. Hence, their increase induces a reduction of the emission quantum yield [72]. Indeed, Φ_PL_ decreases from 92% in crystalline environment to 27% in the less rigid PYD2 matrix (Table 1). Nevertheless, it is attractive to study this emitter material, giving white light luminescence, in a solution-processed OLED.

## 3. Solution-Processed WOLEDs with Cu_2_Cl_2_(P∩N)_2_ as a Single Emitter

**Figure 2:**
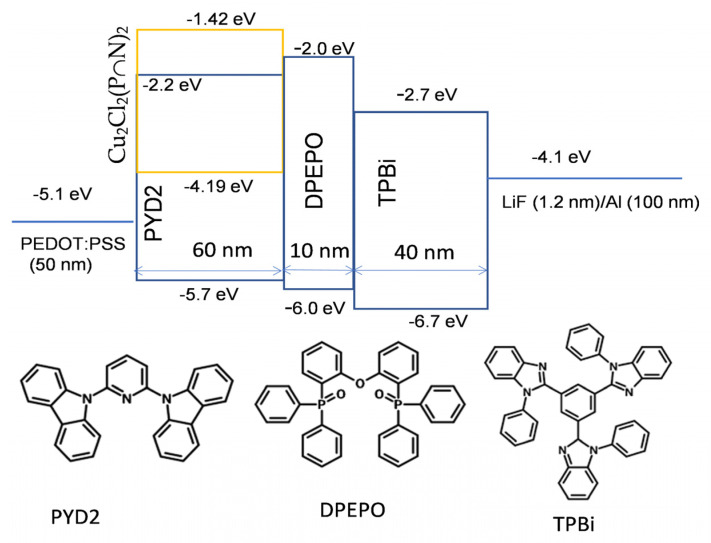
Schematic diagram of the OLEDs, showing the respective energy levels, layer thicknesses, and chemical structures of the organic materials used for the device. The energy levels of Cu_2_Cl_2_(P∩N)_2_ were measured by an electrochemical method and those of other materials were extracted from the literature [72,73,75,76,77].

**Figure 3:**
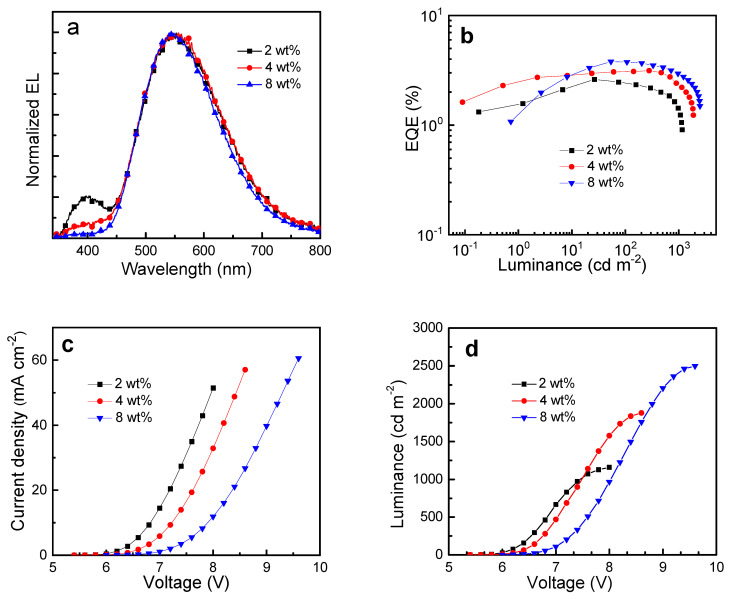
Characteristics of solution-processed devices according to Figure 2 based on Cu_2_Cl_2_(P∩N)_2_ luminescent compounds with concentrations of 2, 4, and 8 wt % in PYD2 host films. (**a**) Normalized EL spectra at 1000 cd m^−2^, (**b**) EQE vs. luminance (**c**) current density vs. voltage, and (**d**) luminance vs. voltage characteristics.

Photoluminescence quantum yield (PLQY) and emission decay time of Cu_2_Cl_2_(P∩N)_2_ in various thin films were measured and the results are summarized in Table 1. PLQY of 27% in PYD2 is the highest yield among the hosts that are frequently applied in solution-processed OLEDs. In fact, PYD2 has been proved as a suitable host for Cu complexes in solution-processed OLEDs [72,73]. Based on this result, solution processed WOLEDs were fabricated using Cu_2_Cl_2_(P∩N)_2_ as a single emitter and PYD2 as host material for the emissive layer (EML). As shown in Figure 2, the device structure was ITO/PEDOT:PSS (50 nm)/PYD2: Cu_2_Cl_2_(P∩N)_2_ (60 nm)/DPEPO (10 nm)/TPBi (40 nm)/LiF (1.2 nm)/Al (100 nm). The layer of poly(3,4-ethylenedioxythiophene) polystyrene sulfonate (PEDOT:PSS) was used between the ITO anode and the EML as a hole-injection layer. The layers bis[2-(diphenylphosphino)phenyl]ether oxide (DPEPO) and 1,3,5-tris(1-phenyl-1H-benzo[d]imidazol-2-yl)benzene (TPBi) function as hole/exciton blocking and electron transporting materials, respectively. As shown in Figure 2, the low-lying LUMO of TPBi facilitates the electron-transporting while the wide band gap of DPEPO can effectively confine excitons inside the EML. Cu_2_Cl_2_(P∩N)_2_ was used as a single emitting dopant in the EML with various concentrations ranging from 2 to 8 wt %. Device performances are depicted in Figure 3, and key data are summarized in Table 2. At a lower concentration of 2 wt %, distinct emission from the PYD2 host peaking at ≈400 nm (Figure 3a) suggests insufficient energy transfer from the host to the emitter, leading to relatively low efficiency, as displayed in Figure 3b. With increasing dopant concentration, the host emission gradually vanishes, and accordingly the efficiency is improved. In addition, as depicted in Figure 3c,d, both current density and luminance significantly decrease with increasing dopant concentration at a given driving voltage, suggesting that charge-trapping could play an important role in these OLEDs [74]. As shown in Figure 2, the high-lying HOMO of Cu_2_Cl_2_(P∩N)_2_ could effectively trap holes injected from the PEDOT:PSS layer. The efficiency of Cu_2_Cl_2_(P∩N)_2_-based devices is limited by the relatively low PLQY of thin films, which is slightly dependent on the dopant concentration. With concentration increase from 4 to 8 wt %, PLQY increases from 19% to 27% (at λ_exc_ = 360 nm), probably because the increased doping concentration leads to an increase of the environment rigidity. Nonetheless, the simple device structure and the ultra-broad electro-luminescence (EL) spectra with full width at half maximum (FWHM) of 153 nm make these studies attractive. One obtains a yellowish white emission with CIE coordinates of (0.38, 0.49) and a color rendering index (CRI) of 64 in the device with 8 wt % of Cu_2_Cl_2_(P∩N)_2_. With the participation of the host emission, the device with 2 wt % Cu_2_Cl_2_(P∩N)_2_ gives an improved white color with CIE coordinates of (0.38, 0.45) and CRI of 72. By improving the PLQY of the films and by further optimizing the device structure, dimeric copper(I) emitters featuring combined TADF and phosphorescence may find wide application in future low-cost WOLEDs.

## 4. Conclusions

In this study, we report on OLED characteristics of the Cu(I) dimer Cu_2_Cl_2_(P∩N)_2_ that features both phosphorescence and TADF at ambient temperature. With the device structure described in Figure 2, we determined an external quantum efficiency of EQE = 3.80 showing warm white emission (CIE coordinates (0.38, 0.49)). This EQE value is lower than expected from the high photoluminescence quantum yield of 92% found for the powder material. However, doping Cu_2_Cl_2_(P∩N)_2_ in the less rigid PYD2 host material allows for more distinct geometry reorganization upon excitation than for the complex in the powder environment. As a consequence, the host with 8 wt % doping concentration exhibits only φ_PL_ = 27% photoluminescence. If we formally normalize EQE to φ_PL_ = 100%, we find EQE(normalized) = 14%. This shows that more efficient devices based on Cu(I) emitters can be obtained, if the emitter’s molecular structure and the host environment can be designed distinctly more rigidly (compare ref. [51]).

## 5. Patents

Parts of this work are related to (i) Yersin, H.; Monkowius, U. Komplexe mit kleinen Singulett-Triplett-Energie-Abständen zur Verwendung in opto-elektronischen Bauteilen (Singulett-Harvesting-Effekt). German Patent DE102008033563 A1, 2008, and (ii) Monkowius, U.; Hofbeck, T.; Yersin, H. Singulett-Harvesting mit zweikernigen Kupfer(I)-Komplexen für opto-elektronische Vorrichtungen. German Patent DE102011080240 A1, 2013.

## Figures and Tables

**Table 1:** Photophysical data of Cu_2_Cl_2_(P∩N)_2_ measured at 300 K.

Photophys. Data	Neat Powder ^(a)^	Doped in PYD2 ^(b)^	Doped in mCP ^(b),(c)^	Doped in PVK ^(b),(c)^	Doped in TCTA ^(b),(c)^	Doped in CBP ^(b),(c)^
λ_max_ ^(d)^	485 nm	544 nm	535 nm	545 nm	542 nm	537 nm
φ_PL_ ^(e)^	92%	27%	20%	11%	10%	13%
τ ^(e)^	8.3 µs	3.1 µs	5.5 µs	3.2 µs	4.3 µs	2.8 µs
∆(S_1_ − T_1_) ^(f)^	930 cm^−1^ (115 meV)					

^(a)^ Data from ref. [48]. ^(^^b)^ This work, doping concentration 8 wt %. ^(c)^ The various host materials are specified in the Appendix A. ^(d)^ Emission maxima. ^(e)^ Photoluminescence quantum yield and emission decay time, respectively. ^(f)^ TADF activation energy.

**Table 2:** Key performances of OLEDs with Cu_2_Cl_2_(P∩N)_2_.

Concentration (wt %)	L ^(a)^ (cd m^−2^)	CE ^(b)^ (cd A^−1^)		PE ^(c)^ (lm W^−1^)		EQE ^(d)^ (%)		CIE ^(e)^ (x, y)	FWHM ^(f)^ (nm)	CRI ^(g)^
		Max	at 1000 cd m^−2^	Max	at 1000 cd m^−2^	Max	at 1000 cd m^−2^			
2	1160	6.48	3.54	3.39	1.48	2.61	1.42	0.38, 0.45	162	72
4	1880	8.39	6.24	4.01	2.71	3.14	2.31	0.38, 0.48	159	69
8	2500	10.5	8.15	4.25	3.20	3.80	2.95	0.38, 0.49	153	64

^(a)^ Maximum luminance; ^(b)^ current efficiency; ^(c)^ power efficiency; ^(d)^ external quantum efficiency; ^(e)^ CIE coordinates at 1000 cd m^−2^; ^(f)^ full width at half maximum at 1000 cd m^−2^; ^(g)^ color rendering index at 1000 cd m^−2^.

## Appendix A

Materials: Cu_2_Cl_2_(P∩N)_2_ was synthesized as described in ref. [48]. PEDOT:PSS [poly(3,4-ethylenedioxythiophene):poly(styrene sulfonic acid)] (Clevios P AI 4083) was purchased from Heraeus; PYD2, DPEPO, TPBi, poly(9-vinylcarbazole) (PVK), 1,3-Bis(carbazol-9-yl)benzene (mCP), 4,4′,4-Tris(carbazol-9-yl)triphenylamine (TCTA), 4,4′-Bis(carbazol-9-yl)biphenyl (CBP), and LiF from Luminescence Technology Corp; Aluminum pellets from Kurt J. Lesker. All materials were used as received.

Photophysical measurement (see Table A1): Thin-film samples of Cu_2_Cl_2_(P∩N)_2_ doped in PYD2, mCP, PVK, TCTA, and CBP, respectively, were prepared by drop-cast from a chlorobenzene solution containing Cu_2_Cl_2_(P∩N)_2_ (8 wt %). The solvent was evaporated at 80 °C and translucent films were obtained. PLQYs of these thin-film samples were measured with the Hamamatsu C11347 Quantaurus-QY absolute PL quantum yield measurement system. Emission lifetime measurements were performed on a Quanta Ray GCR 150-10 pulsed Nd:YAG laser system.

**Table A1:** Electrochemical properties of Cu_2_Cl_2_(P∩N)_2_.

	*E*_ox_ and *E*_red_ (V) ^(a)^	*E*_HOMO_ and *E*_LUMO_ (eV) ^(b)^
Cu_2_Cl_2_(P∩N)_2_	0.504; −2.488	−4.91; −1.42

^(a)^ Values obtained from differential pulse voltammetry measurements which were carried out in acetonitrile with 0.1 mol·dm^−3^ [*n*Bu_4_N]PF_6_ as supporting electrolyte and saturated calomel electrode (SCE) as the reference electrode with a scan rate of 100 mV·s^−1^. Potentials reported here versus SCE. ^(b)^ The HOMO and LUMO energy levels were estimated based on the equations of *E*_HOMO_ = −(1.15 × *E*_ox_ + 4.79) eV, *E*_LUMO_ = −(1.18 × *E*_red_ + 4.83) eV [19,78]; the potential *E*_ox_ of ferrocene is 0.40 V.

Fabrication process of solution-processed OLEDs: An aqueous solution of PEDOT:PSS was spin-coated onto the cleaned ITO coated glass substrate and baked at 120 °C for 20 min to remove the residual water solvent in a clean room. Afterwards, the mixture of PYD2 and the emitting dopant in chlorobenzene was spin-coated atop the PEDOT:PSS layer inside the glove box. After annealing at 70 °C for 30 min, all devices were subsequently transferred into a Kurt J. Lesker SPECTROS vacuum deposition system without exposing to air. In the vacuum chamber, organic materials of DPEPO and TPBi were thermally deposited in sequence at a rate of ~0.5 nm s^−1^. Finally, LiF (1.2 nm) and Al (100 nm) were thermally deposited at rates of 0.03 and 0.2 nm s^−1^, respectively.

Characterization of OLEDs: Current density-brightness-voltage characteristics, EL spectra, and EQE of EL device were obtained by using a Keithley 2400 source-meter and an absolute external quantum efficiency measurement system (C9920-12, Hamamatsu Photonics).
